# Modeling and simulation of a Tamil language encoder for advanced encryption technologies

**DOI:** 10.1016/j.patter.2023.100740

**Published:** 2023-05-03

**Authors:** Shan Suthaharan

**Affiliations:** 1 UNC Greensboro, Greensboro, NC 27402, USA

**Keywords:** Tamil language, Galois field, encryption, pattern recognition, confidentiality, Hardy-Ramanujan number, planets, Shannon’s idea, confusion, diffusion

## Abstract

This paper presents a Tamil language (TL) encoder that benefits advanced encryption technologies such as the advanced encryption standard (AES). It defines a product set of vowel and consonant sounds of the Tamil language and reveals its connection to Hardy-Ramanujan prime factors and Tamil letters as a one-to-one mapping. It also reveals that the Tamil letters, combined with the digits from 1 to 9, form a Galois field of $2^{8}$ over an irreducible polynomial of degree 8. Additionally, it implements these two mathematical properties, models the TL encoder, and replaces the pre-round transformation of AES with the model to enhance cryptographic strengths. The cryptographic strengths are measured by the runs test scores of the bit sequences of the ciphers of AES and compared with that of English language. The modeling and simulation conclude that the TL encoder enhances the cryptographic strength of AES at every step of the encryption flow.

## Highlights

• A connection between Hardy-Ramanujan prime factors and Tamil letters is reported
• A Tamil language encoder is proposed to replace the pre-round transformation of AES
• Tamil language can provide a stronger cryptographic strength than English language
• Tamil language encoder can enhance the strengths of AES for data science applications

## The bigger picture

The misuse of modern technology is a huge threat to the privacy of data, the safety of machine learning models, and the security of encryption techniques. With advances in natural language processing—one of the areas of artificial intelligence—it is possible to utilize the cryptographic features of other natural languages (other than English language) to enhance the cryptographic strengths of current encryption techniques. Every language has unique features; however, in this paper, the Tamil language (TL) is considered and utilized to develop a TL encoder to enhance the cryptographic strength of the advanced encryption standard (AES). The establishment of a one-to-one mathematical relationship between the Hardy-Ramanujan prime factors and Tamil letters, and the formulation of a TL-based set that satisfies the properties of Galois field (finite field), can deliver a significant impact to the short- and long-term goals (privacy, safety, and security) of data science and machine learning.

## Graphical abstract

**Figure:**
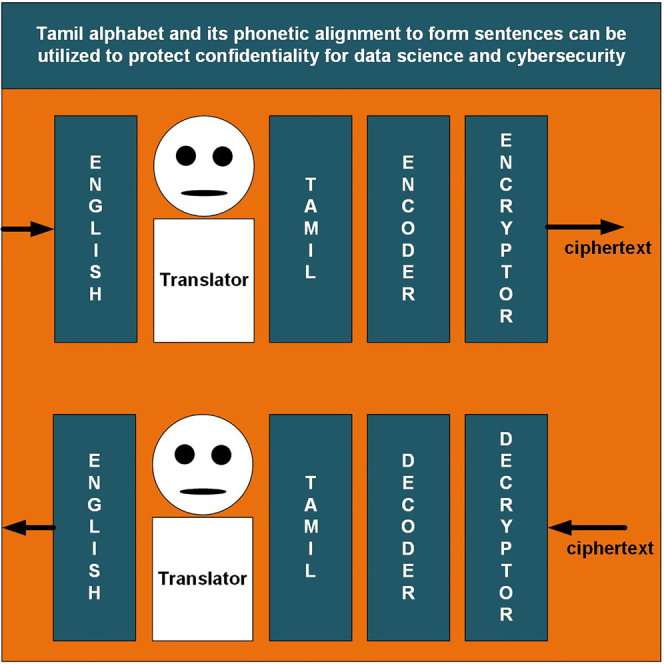

## Teaser

The hidden mathematical properties of Tamil language have been acknowledged. It has been shown that these properties can be utilized to enhance the cryptographic strengths of the advanced encryption standard. The revelation of these mathematical structures can also contribute to the advances of secure natural language processing—one of the concepts of artificial intelligence.

## Introduction

The Tamil alphabet and its phonetic alignment to form sentences have been utilized to develop robust confidentiality protection techniques for cybersecurity. Confidentiality protection is one of the essential requirements in cybersecurity. The advanced encryption standard (AES) is an algorithm that is currently standardized and widely used in many cybersecurity applications. The AES algorithm first encodes a message to hexadecimal states and then performs several encryption tasks that generate confusion and diffusion properties—the two major cryptographic properties defined based on Shannon’s idea.^1^ Hence, message encoding is one of the essential tasks in the encryption processes; however, the encoding of English texts using positional information of the letters in the alphabet develops vulnerability at that step, which then may propagate through the remaining encryption steps of the AES algorithm. The English alphabet, with only a set of 26 letters, generates significant redundancy; hence, it is difficult to develop robust encoding techniques. Therefore, it is important to study this problem using other languages by leveraging their scientific properties. The Tamil language is a perfect candidate for this purpose; hence, its cryptographic properties have been studied in this article. It is interesting to learn how the vowels and consonants are named in Tamil. The vowels are called Uyir Ezhuthu, meaning the soul letters, and the consonants are called Mei Ezhuthu, meaning the body letters. One way to interpret this is that the vowels give a soul to the context of the Tamil language, while the consonants give a body to the context. However, another way of interpreting this may be that the Tamil vowels sounds—when spoken with proper pronunciation—give benefits to the soul and the consonants sounds give benefits to the body, which, in turn, may give many health benefits by enhancing circadian rhythms (this is just a hypothetical statement). It is also interesting to learn how other letters in the Tamil alphabet are formed by combining the vowels and consonants, which are called UyirMei Ezhuthu, meaning soul+body letters.

The Tamil language—while providing a phonetic expressive communication—may bring scientific benefits to many applications that include natural language processing, information security, and artificial intelligence. It carries many hidden computational constituents that must be studied and understood in scientific perspectives to be adapted in modern applications. For instance, the power of the divine sound and the vibration of the word ஓம் (ōm) must be studied in depth to understand its healing power. The word ஓம் is regularly used by Tamils (in particular by Tamils in Sri Lanka) in daily dialogue to express an agreement that provides the same meaning as the word “yes” in English dialogues. The well-known Indologist, Asko Parpola, also mentioned in his book that the word ஓம் contributes to Dravidian languages.^2^ More information about his descriptions and discussions on the word ஓம் can be found on page 170 of his book.^2^ Hence, the Tamil language, if properly spoken, may also bring health benefits to the speakers of the Tamil language. Today, we can witness the contribution of the ஓம் sound to physical and mental health through yoga in the modern society; however, its scientific significance is still to be explored in detail.

The Tamil language occupies the $17^{th}$ position in the top 200 most spoken languages in the world—according to the current statistics available at https://www.ethnologue.com/guides/ethnologue200 (accessed online on January 9, 2022). This website also informs us that Tamil is spoken by 85 million people in the world. The first top 20 languages are as follows: English (1,348 M), Mandarin Chinese (1,120 M), Hindi (600 M), Spanish (543 M), Standard Arabic (274 M), Bengali (268 M), French (267 M), Russian (258 M), Portuguese (258 M), Urdu (230 M), Indonesian (199 M), Standard German (135 M), Japanese (126 M), Marathi (99 M), Telugu (96 M), Turkish (88 M), Tamil (85 M), Yue Chinese (85 M), Wu Chinese (82 M), and Korean (82 M). Among these top 20 languages, Tamil may be the only language that has a number of letters in its alphabet that is closer to 256 such that it can meaningfully and securely occupy an 8-bit processor in a computing device. The 8-bit processor is a key player in many secure storage and computing applications in computer science that include cryptography and network security applications and secure reconfigurable hardware applications.^3^^,^^4^^,^^5^

The paper^6^ also states that a large number of people from several countries that include Fiji, India, Malaysia, Mauritius, Singapore, South Africa, and Sri Lanka speak Tamil. In the last three decades, a large number of Tamils have migrated to Australia, Europe, and North America; hence, it is expected that this number could be higher. Therefore, an advancement in Tamil language research can benefit its globalization. In recent years, there has been a growing interest in its applications to cryptography,^7^^,^^8^ speech recognition,^9^ character recognition,^10^^,^^11^ and semantics analysis.^12^ Most recently, mental health research, related to the pandemic, has also been performed in the Tamil population, where the Tamil language could have an indirect influence in the study,^6^^,^^13^ in addition to the cultural and geographical contributors.

The focus of this article is the application of the Tamil language to cryptography. The Tamil language has many hidden patterns that might be useful for cryptographic applications. With the reduction in redundancy—caused by the large number of letters in the alphabet—the Tamil language can provide stronger confidentiality protection than the English language when applied to encrypt messages and share them over the Internet. In Geetha et al.,^8^ for example, it has been stated that the 247 letters in the Tamil alphabet make it difficult to crack a Tamil message hidden in a cipher. They mapped (encoded) translated Tamil texts to a randomly selected 2-bit combination of English letters and used the AES algorithms to encrypt the encoded Tamil texts. However, limited studies have been performed to understand the mathematical and scientific properties of the Tamil language and their connections to develop interpretable cybersecurity environment.

The AES helps protect the confidentiality of a block of texts by applying encryption modules.^14^ As the encryption standard, it is widely used in many applications that require confidentiality protection. The AES algorithm adapts the concept of a Galois field of $2^{8}$ with an irreducible polynomial of degree 8. It takes a block of characters and encrypts it through sequentially established mathematical transformations while adding a set of keys so that the plaintext of a ciphertext cannot be recovered by an adversary. In essence, it uses multiple rounds of encryption where each round has a set of transformations that include Sub-Bytes, ShiftRows, MixColumns, and AddRoundKey to achieve its encryption goals. These transformations are operated on a Galois field of $2^{8}$ with an irreducible polynomial, multiplication and addition operators, and multiplicative inverse, hence delivering cryptographic strengths to the encryption flow. The AES algorithm, when applied to Tamil texts, is expected to provide stronger ciphers than English since the Tamil alphabet has much larger number of letters than English. The proposed TL encoder in this paper adds scientific and mathematical properties of the Tamil language to the encryption flow of the AES algorithm.

### Modern encryption

Modern encryption techniques leverage the intrinsic cryptographic properties of a finite field and the Shannon’s idea,.^1^^,^^15^ The 8-bit words and the corresponding integer representations performed in computer applications triggered the use of Galois field GF $\left(2^{8}\right)$ with an irreducible polynomial of degree 8.^14^^,^^16^ They also contributed to the cryptographic strength of the AES algorithm via Galois field operations, the multiplicative inverse, the add round keys module, and the confusion and diffusion properties.

### Galois field

A Galois field is a set of n-bit (n>0) integers together with two operation additions and multiplication that are defined over an irreducible polynomial of degree n that satisfies the following axioms of a finite field^15^: closure under addition, associative law of addition, commutative law of addition, existence of additive identity, existence of additive inverse, closure under multiplication, associative law of multiplication, commutative law of multiplication, distributive law, existence of multiplicative identity, existence of multiplicative inverse, and no zero dividers. The mathematical operations in the Galois field are generally represented by addition and multiplication tables along with additive and multiplicative inverses while satisfying the aforementioned axioms. The readers are encouraged to refer to the scientific materials in Dodis et al.,^1^ Stallings,^15^ and Forouzan^17^ to obtain detailed information. It is well understood that each element of the Galois field appears the same number of times in the addition and multiplication tables (except the zero in the multiplication table), and this property makes the Galois field a perfect mathematical tool for modern cryptographic systems. In other words, this property of the Galois field notably contributes to the security features of the AES because the probability of the occurrence of each integer for an operation over a Galois field is the same, and it is equal to $1/2^{n}$, where n=8 for the AES. For a large value of n, this probability is very small, and consequently, the cryptographic strength becomes stronger. The AES algorithm is one of the most popular encryption algorithms that are currently used by the industry and government agencies.

### AES algorithm

In the AES algorithm, the Galois field of 8-bit integers represents a finite field that consists of 256 (i.e., $2^{8}$) 8-bit integers 0,1,2,…,255 and two arithmetic operations (addition and multiplication) on the 8-bit integers that are represented by a pair of hexadecimal numbers (00, 01, 02, …, FF) and defined over an irreducible polynomial $R\left(x\right):x^{8}+x^{4}+x^{3}+x+1$ of degree 8.^18^ There are 30 possible irreducible polynomials attached to an 8-bit integer.^19^ As stated in Stallings,^15^ the developers of the AES have selected the first irreducible polynomial on the list to construct the addition and multiplication tables and the additive and multiplicative inverses. The AES algorithm, as stated earlier in this article, assumes that each byte (8-bits) of its input bits (plaintext that is the message to be encrypted) is an element in the Galois field of 8-bit integers in which the addition and multiplication operations are defined over the irreducible polynomial $R\left(x\right):x^{8}+x^{4}+x^{3}+x+1$ of degree 8. It is evident from the design of the AES algorithm^14^^,^^15^ that the AddRoundKey operation is added to each round to make the inverse transformation highly resistant to the adversarial attacks that attempt to recover plaintext from a given ciphertext. In this article, our goal is not to develop another encryption technique but rather to show the cryptographic strengths of the Tamil language that could support the AES algorithm to become much stronger with fewer numbers of rounds and intermediate encryption.

## Results

**Figure 1:**
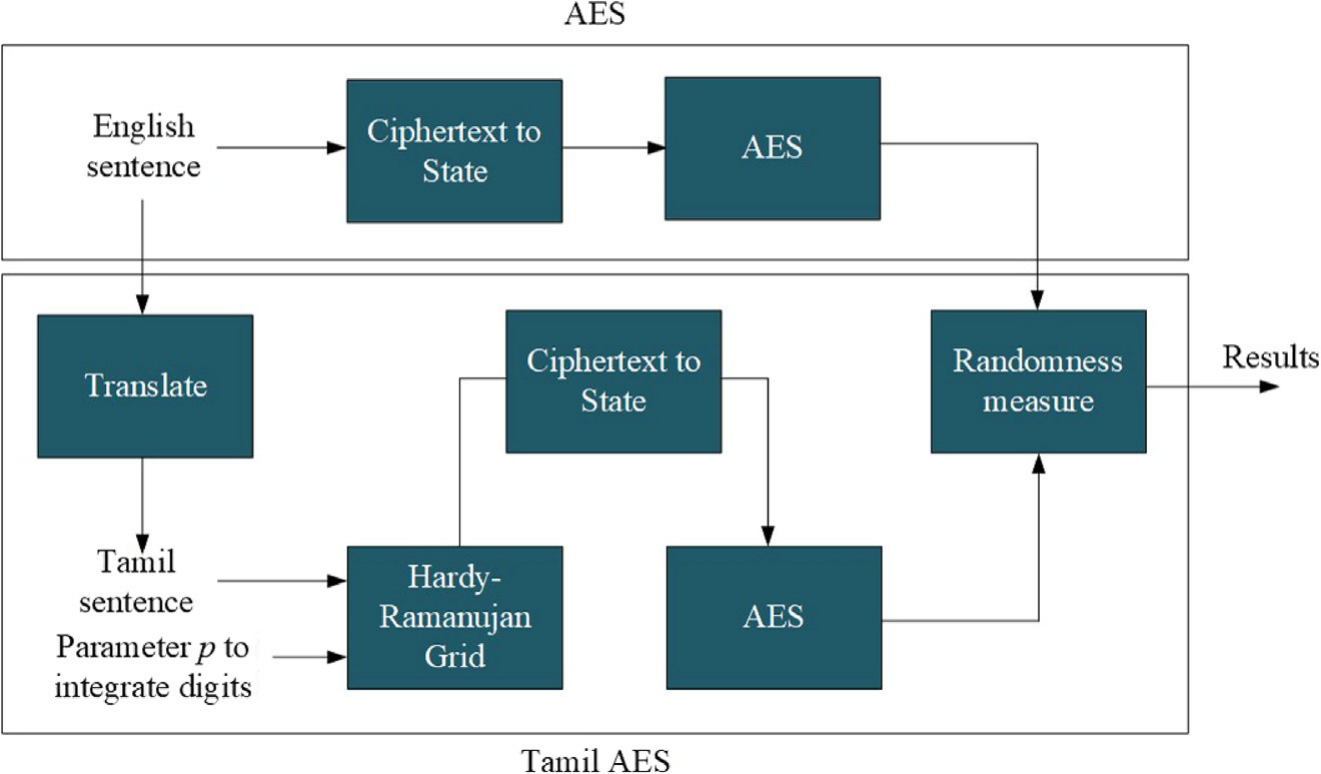
The proposed computational framework The placement of the elements of the APT-Grid on the GFT-Grid in Figure 5 is parameterized by p in this framework such that it can be used for message authentication and integrity in addition to the establishment of the confidentiality protection.

**Figure 2:**
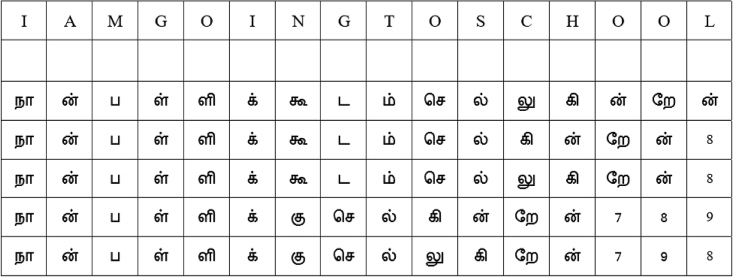
The English and Tamil text examples used in the experiments Note that this is a plaintext example that shows multiples ways of writing an English sentence in Tamil. Hence, the integers 7, 8, and 9 that are used to fill the holes are also plaintext, and they will be encoded to BA, D9, and F8 based on the GFT-Grid in Figure 5.

In our experimental analysis, using the proposed computational framework, we studied the effects of English and Tamil plaintexts at the intermediate and the final steps of the AES algorithm without using the round keys. The flow of analysis that is presented in Figure 1 is adapted to systematically perform the experiments. We know that the use of the round keys provides significant protection against attacks that attempt to recover the plaintext from a given ciphertext. In our experiments, we are only interested in the effect of plaintexts (English and Tamil) in developing randomness in the intermediate and the final ciphertexts and the confidentiality protection of the AES modules. The English and Tamil plaintexts in Figure 2 are used.

### Runs test validation

The cryptographic strength of the proposed TL encoder is validated using the runs test and compared with that of the English-based encryption approach. The runs test measure has been widely used in statistical process analysis to determine the randomness of bit sequences.^20^ It defines a testing hypothesis ($H_{0}$: a sequence is random) with an alternative hypothesis ($H_{1}$: the sequence is not random) and develops the runs test measure ρ as follows^20^^,^^21^:

and (Equation 1) $$\mu =\frac{2n_{1}n_{2}}{n_{1}+n_{2}}+1\;\sigma =\sqrt{\frac{2n_{1}n_{2}\left(2n_{1}n_{2}-n_{1}-n_{2}\right))}{{\left(n_{1}+n_{2}\right)}^{2}\left(n_{1}+n_{2}-1\right)}}\text{,}$$

(Equation 2) $$\rho =\frac{\left|r-\mu \right|}{\sigma }\text{,}$$

where μ is the expected number of runs in the bit sequence, σ is the standard deviation of the number of runs, $n_{1}$ and $n_{2}$ are the numbers of zeros (0) and ones (1) in the bit sequence, and the variable r represents the number of runs found in the sequence. As stated in NIST,^21^ we can reject the null hypothesis, $H_{0}$: a sequence is random with 95% confidence if ρ>1.96, provided $n_{1}$ and $n_{2}$ are greater than 10. We can use the deviation between ρ and 1.96 as a measure of cryptographic strength to compare the performance of English and Tamil encryptions. The non-random bit sequences in a ciphertext can expose the deterministic patterns, which could be used by adversaries to recover its plaintext by using a brute force attack, as an example. Two coding (Python) examples that use the runs test to validate the randomness of English and Tamil texts are given in Figures S1 and S2. The outputs of these Python modules are presented at the bottom of each listing as comments. A significant randomness, as diffusion, can be seen in the placement of 0s and 1s in the Tamil-encoded text compared with that of the English text.

### Experiment 1

**Figure 3:**
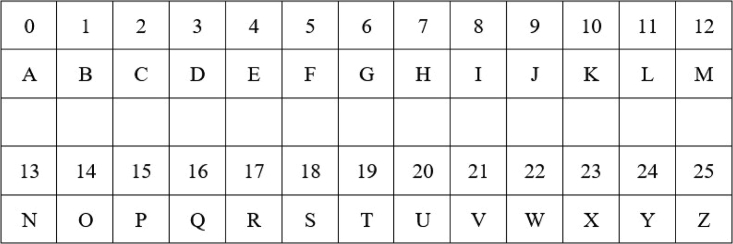
In the AES algorithm, the letters in the English alphabet are encoded by using their location profiles in the English alphabet This figure illustrates the assigned numbers for the letters in the English alphabet in the ordered list: the letter “A” is assigned to the location index “0,” “B” is assigned to the location index “1,” …, “Z” is assigned to the location index “25.”^15^^,^^17^

**Figure 4:**
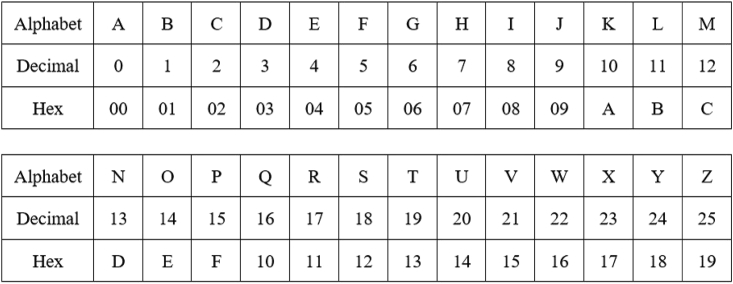
An encoder for the English alphabet (decimal to hex)^17^

The English sentence “I am going to school” in Figure 2 is used in this experiment along with the flow of analysis at the top stream of Figure 1. As traditionally performed in the AES algorithm, this sentence is encoded to hexadecimal form using the location profiles of the letters in the English alphabet. Figure 3 shows the location information, and Figure 4 shows their hexadecimal representations. The values in Figure 4 are used for encoding the English text for the pre-round transformation of the AES. The encoded text, in its bit sequence form, is analyzed to measure the level of randomness using the runs test score ρ. The results are presented in the first row of Table 1. The runs test score of 3.102 (i.e., above 1.96) indicates that the resulting encoded text at the pre-round step is not random for the English plaintext “I am going to school.” The encoded text is then used to generate the ciphertexts (without using the round keys) by applying the Sub-Bytes, ShiftRows, and MixColumns transformations in the encryption flow of the AES algorithm. The randomness of the ciphertexts of these transformations is then measured by calculating the runs test scores as previously performed. The results are, respectively, presented in the first row of Tables 2, 3, and 4. As we can observe, the Sub-Bytes transformation achieves randomness with a runs test score of 1.045, the ShiftRows transformation achieves randomness with a runs test score of 1.045, and the MixColumns transformation achieves randomness with a runs test score of 1.559. Hence, the addition of these transformations provides an increased cryptographic strength to the English-based AES encryption.

**Table 1:** Comparison of cryptographic strength after pre-round transformation

Plaintext	Mean, μ	SD, σ	Runs test score, ρ	Random (Y/N)
English sentence	51.859	4.468	3.102	N
Tamil sentence 1	64.609	5.600	0.287	Y
Tamil sentence 2	64.438	5.585	0.078	Y
Tamil sentence 3	64.609	5.600	0.109	Y
Tamil sentence 4	63.438	5.496	0.079	Y
Tamil sentence 5	63.734	5.222	0.048	Y

**Table 2:** Comparison of cryptographic strength after Sub-Bytes transformation

Plaintext	Mean, μ	SD, σ	Runs test score, ρ	Random (Y/N)
English sentence	62.359	5.400	1.045	Y
Tamil sentence 1	65.000	5.635	0.000	Y
Tamil sentence 2	65.000	5.635	0.355	Y
Tamil sentence 3	64.984	5.633	0.003	Y
Tamil sentence 4	63.438	5.496	0.079	Y
Tamil sentence 5	64.984	5.633	0.003	Y

**Table 3:** Comparison of cryptographic strength after ShiftRows transformation

Plaintext	Mean, μ	SD, σ	Runs test score, ρ	Random (Y/N)
English sentence	62.359	5.400	1.045	Y
Tamil sentence 1	65.000	5.635	0.355	Y
Tamil sentence 2	65.000	5.635	0.887	Y
Tamil sentence 3	64.984	5.633	1.068	Y
Tamil sentence 4	64.938	5.496	0.366	Y
Tamil sentence 5	64.984	5.633	0.358	Y

**Table 4:** Comparison of cryptographic strength after MixColumns transformation

Plaintext	Mean, μ	SD, σ	Runs test score, ρ	Random (Y/N)
English sentence	64.750	5.612	1.559	Y
Tamil sentence 1	64.000	5.546	1.082	Y
Tamil sentence 2	64.938	5.629	0.189	Y
Tamil sentence 3	64.984	5.633	0.003	Y
Tamil sentence 4	64.000	5.546	0.180	Y
Tamil sentence 5	64.984	5.633	0.713	Y

### Experiment 2

**Figure 5:**
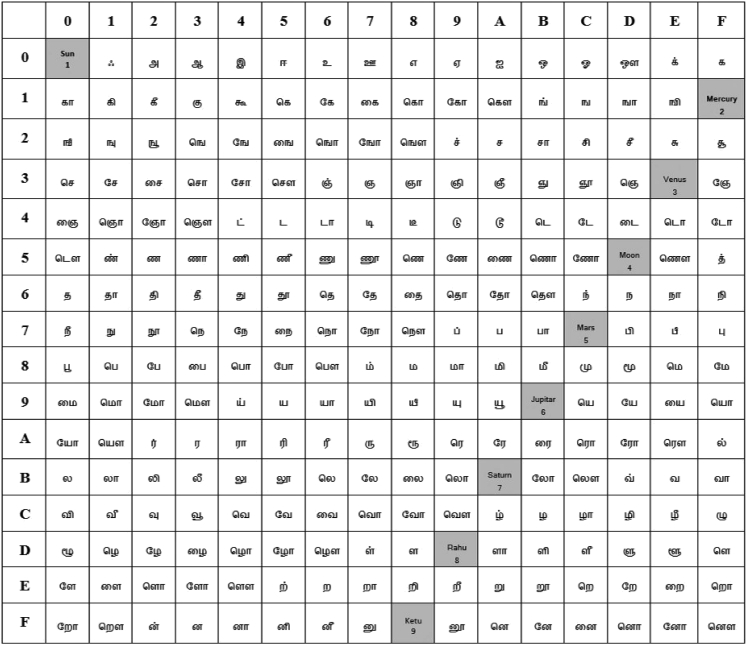
The proposed GFT-Grid that comprises the HRT-Grid and APT-Grid Note that the hex numbers (rows and columns) are assigned purely based on the 8-bit numbering rather than the actual Unicode numbering assigned for Tamil letters or the actual characterization incorporated in the Unicode. However, the concept can be easily translated to the Unicode system with modifications. The placement of the APT-Grid on the GFT-Grid is parameterized.

**Figure 6:**
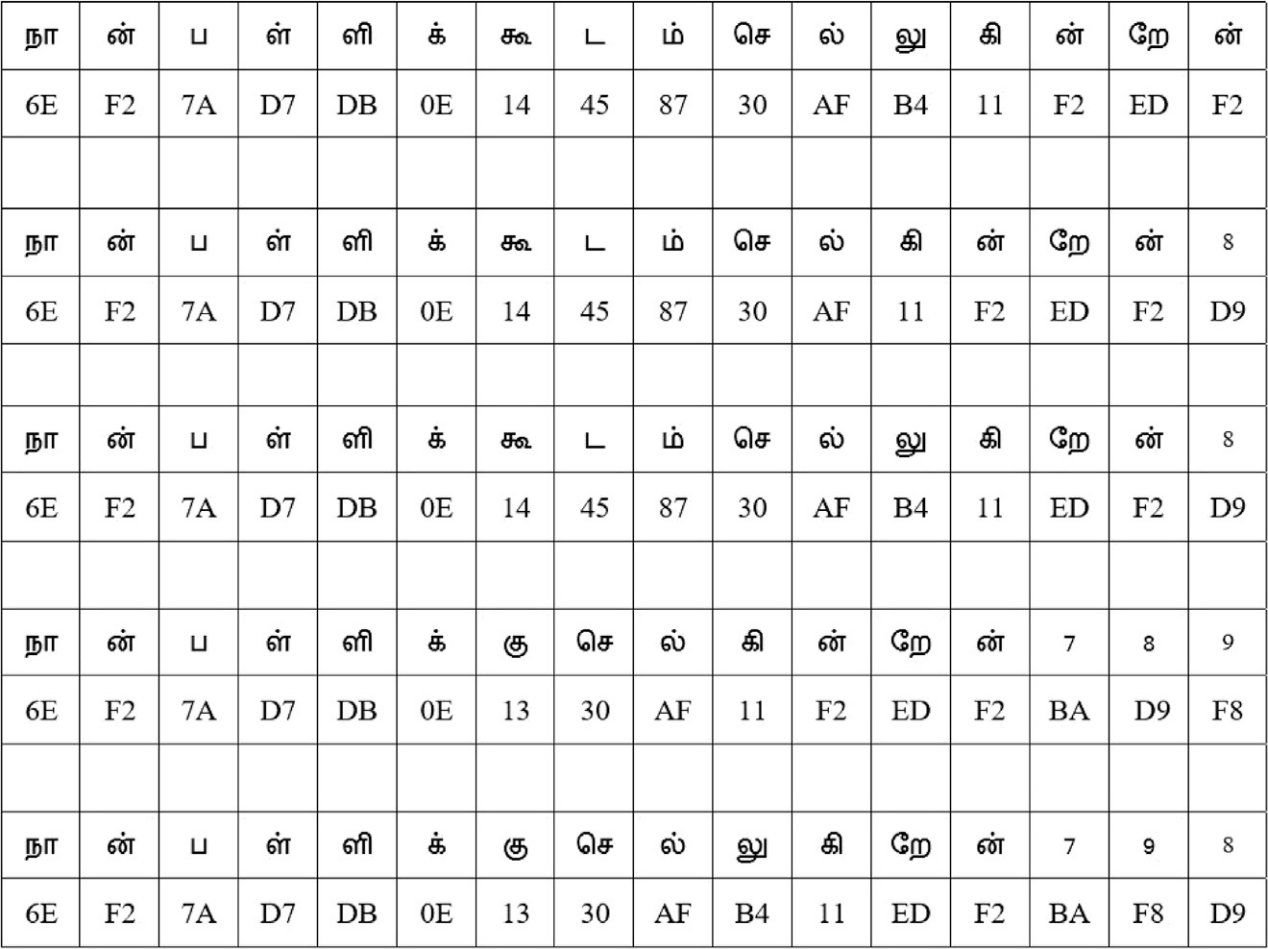
Five versions of the Tamil texts that provide the same message as “I am going to school” This figure shows the encoded versions of the five texts using the GFT-Grid that satisfies the Galois field GF $\left(2^{8}\right)$. Note that the digits from the APT-Grid are selected empirically and added as padding to the encoded Tamil texts; however, these padded sequences can be used to authenticate the message or confirm the integrity of the message in addition to their contribution to confidentiality protection.

The translated Tamil sentences of the English sentence “I am going to school” in Figure 2 are used in this second experiment along with the flow of analysis that is presented at the bottom stream of Figure 1. The encoded versions of the Tamil texts using the Galois field of the Tamil language—by applying the Galois field Tamil grid (GFT-Grid) in Figure 5—are shown in Figure 6. All the encoded Tamil texts, in their bit sequence forms, are then analyzed, and the level of randomness is measured by using the runs test score ρ. The results are presented in the second through sixth rows of Table 1. All five Tamil texts pass the randomness tests for the pre-round transformation of the AES algorithm with lower scores that range from 0.048 to 0.287 and an average score of 0.1202, which is significantly lower than the runs test score of the English text (3.102). Therefore, by comparing all the runs test scores in Table 1, we can say that the Tamil texts carry cryptographic properties that are useful for AES encryption and hence help achieve stronger cryptographic strength at the pre-round transformation stage of the AES encryption algorithm.

The encoded Tamil texts are then used, as previously, to generate the ciphertexts (once again without using the round keys) by applying the Sub-Bytes, ShiftRows, and MixColumns transformations in the encryption flow of the AES algorithm. The randomness of the ciphertexts of these transformations for each Tamil text is then measured by calculating the runs test scores as performed for the English text. The results are, respectively, presented in the corresponding rows of Tables 2, 3, and 4. The results of Sub-Bytes in Table 2 show that the Tamil texts achieve significant randomness with scores that range from 0 to 0.355 and an average score of 0.088. We can also observe that Tamil sentence 1 has achieved a perfect randomness based on the runs test score, while Tamil sentences 3, 4, and 5 achieve very high randomness.

Table 3 shows the results after the application of ShiftRows transformation of the AES encryption flow to the Tamil texts used. The results show that the Tamil texts yield the randomness with runs test scores that range from 0.355 to 1.068 and an average score of 0.6068. Similarly, Table 4 presents the results after the application of MixColumns transformation of the AES encryption flow to the Tamil texts used. The results show that the Tamil texts yield the randomness with runs test scores that range from 0.003 to 1.082 and an average score of 0.4334. Tables 1, 2, 3, and 4 also provide the mean and standard deviations that are used to calculate the runs test scores. The similar standard deviation values indicate that the runs test scores calculated for the English text and the corresponding translated Tamil versions are on similar agreement; hence, the meaning of their scores interpretable in the same way. Hence, by combining the results in these tables, we can conclude that the Tamil text can provide a very high cryptographic strength to the AES encryption flow through its mathematical properties associated with the Galois field GF $\left(2^{8}\right)$.

## Discussion

A summary of the results is presented in Table 5 based on the following linear scale of the runs test scores: the range ρ>1.96 means that the cryptographic strength is very weak (i.e., non-random); the interval 1<ρ≤1.96 means that the cryptographic strength is weak; the interval 0.5<ρ≤1 means that the cryptographic strength is strong; and the interval 0≤ρ≤0.5 means that the cryptographic strength is very strong. The results in every row of Table 5 clearly indicate that the Tamil language provides stronger cryptographic strengths than the English language. Similarly, the results in the second and third columns clearly indicate that the Tamil language provides very strong cryptographic strengths to the pre-round and Sub-Bytes steps of the AES while creating possibly weaker situations in ShiftRows and MixColumns ($4^{th}$ and $5^{th}$ columns of Table 5). However, by combining the strengths in their associated cryptographic modules, the Tamil language can overall provide very strong cryptographic strengths to the encryption flow of the AES. This also suggests that the encryption of the Tamil text with only the pre-round transformation is sufficient to develop a very strong cryptographic strength in the ciphertext. It can also give much stronger cryptographic strength when the round keys are added. When the other rounds with Sub-Bytes, ShiftRows, and MixColumns are added to generate confusion and diffusion, then the Tamil language texts are expected to give remarkable cryptographic strengths to the AES algorithm, based on the results presented in Table 5.

**Table 5:** Summary of cryptographic strengths

Plaintext	Pre-round	Sub-Bytes	ShiftRows	MixColumn
English	very weak	weak	weak	weak
Tamil 1	very strong	very strong	very strong	weak
Tamil 2	very strong	very strong	strong	very strong
Tamil 3	very strong	very strong	weak	very strong
Tamil 4	very strong	very strong	very strong	very strong
Tamil 5	very strong	very strong	very strong	strong

It is also important to note that the proposed APT-Grid has been attributed to the 9 ancient astrological planets, and we utilized them for filling the 9 holes in the GFT-Grid. This is a proposition of the proposed TL encoder. In other words, it is the introduction of a new definition for a way to encode a Tamil language plaintext for the purpose of encryption by mapping the 9 holes onto a set of the 9 well-known ancient astrological planets. When the Hardy-Ramanujan Tamil grid (HRT-Grid) of 13 × 19 is transformed to the GFT-Grid of 16 × 16 for applying the Galois field, 9 holes are created. By filling these gaps with a set of 9 integers or words that are associated with them, they can serve as a secure key. However, to make the algorithm suitable for standardization, a particular plaintext with a set of 9 words (or characters) is required. Since it is well known that there are 9 ancient astrological planets, this can fulfill the requirement for the proposed approach. Hence, a proposition is built in this article to form an APT-Grid of 3 × 3. However, what is required is a set of nine integers (e.g., 1, 2, … 9) to fill the holes in the GFT-Grid, and the users of the TL encoder could select their own secure message for these keyholes.

A cryptographic system that employs an AES-based technology with the proposed TL encoder can resist attacks better than a system that employs just an AES algorithm. Since the addition of TL encoder, as demonstrated in the experimental procedures, increases the cryptographic strengths of the system at every step of the AES flow, it is now possible to use private keys with much smaller lengths. As a result, its application can be easily extended to resource-limited applications that include Internet of Things (IoT) security, sensor network security, and mobile device security. As such, the communication cost in a data communication network can be significantly reduced. On the other hand, a cryptographic application that employs the proposed TL encoder requires a translation device between Tamil and English languages. This is one of the limitations. The other limitation is the additional computational time that is required for encoding and decoding the plaintext and ciphertext at the source and the destination. These limitations—compared with the cryptographic strengths of the encoder as stated above—are negligible.

## Experimental procedures

### Resource availability

#### Lead contact

Please direct your questions and resource requests to the lead contact, Shan Suthaharan (s_suthah@uncg.edu).

#### Materials availability

This study did not generate new unique reagents.

### Proposed procedures

**Figure 7:**
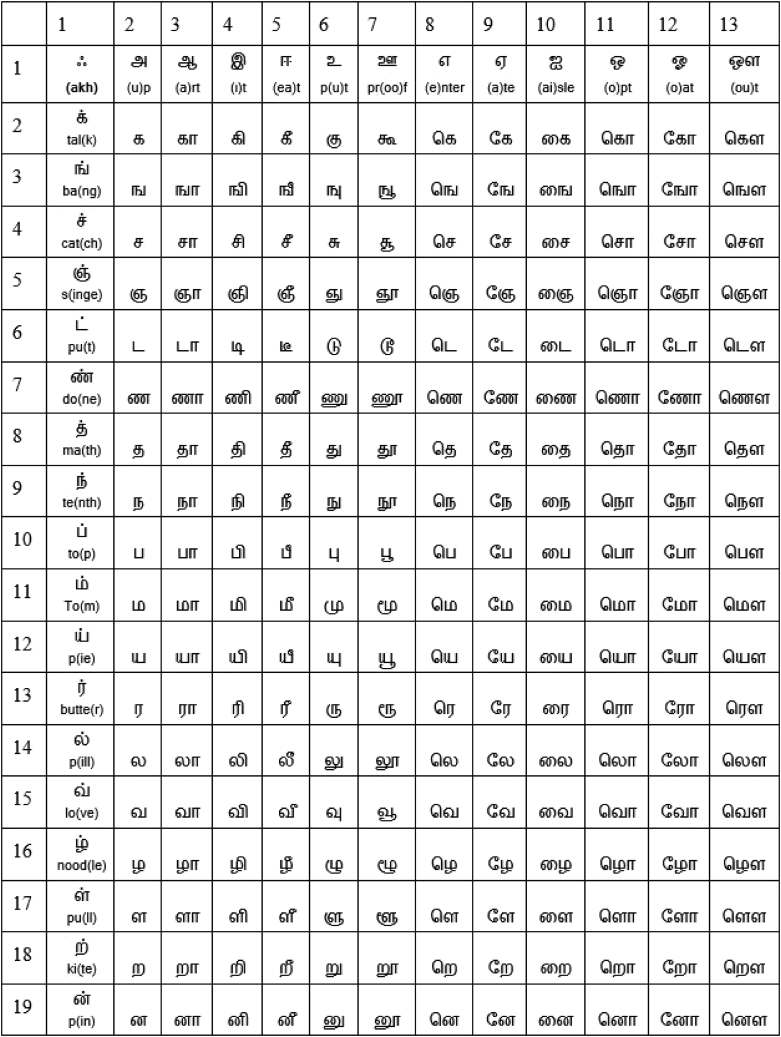
The alphabet of the Tamil language (defined as HRT-Grid in this article) There are 247 Tamil letters that make 13×19 sounds by directly using 12 vowels (first row; columns 2–13), 18 consonants (first column; rows 2–19), and a special letter akh (first cell) and combining them. It also presents the pronunciation for the vowels and consonants, as a guide, to pronounce other letters. Readers are encouraged to refer to Schiffman^24^ for detailed information on Tamil language pronunciation.

The proposed method presents three new definitions, HRT-Grid, astrological planet Tamil grid (APT-Grid), and GFT-Grid, and develops mathematical connections between them. The HRT-Grid is a grid that is formed by the product set of the sounds of vowels and consonants of Tamil letters and that has a dimension of 13×19, where 13 and 19 are the prime factors of the Hardy-Ramanujan number 1,729. Note that the Hardy-Ramanujan number 1,729 proved to have significant mathematical contributions; for example, Ono and Trebat-Leder have shown its connection to the K3 surface.^22^ The APT-Grid is a set with a dimension of 3×3 that consists of nine digits 1,2,…,9, where each digit may represent an astrological planet.^23^ Similarly, the GFT-Grid represents a grid that has a dimension of 16×16, where the elements of the GFT-Grid, which are represented by 8-bit integers, are the letters of the Tamil alphabet. The proposed method also presents two mathematical modeling approaches and one simulation architecture. The first modeling component defines a one-to-one mapping between the HRT-Grid and the Tamil alphabet, as shown in Figure 7. The second modeling component defines a one-to-many mapping between the Tamil alphabet (247 letters) and the 16×16 (= 256) elements of the GFT-Grid such that the elements of this square grid satisfy the properties of a Galois field of GF $\left(2^{8}\right)$ that plays a major role in the AES algorithm.^15^ The APT-Grid is used to parameterize the elements of the GFT-Grid and systematically complete the 9 holes in the GFT-Grid.

The proposed simulation architecture presents a computational framework that helps us model and analyze the HRT-Grid and GFT-Grid properties and evaluate the cryptographic strength of the Tamil language using the AES algorithm and the runs test scores as the measure of randomness. In other words, the transformation of the contextual properties of the Tamil alphabet to a mathematical measurement domain is modeled by leveraging the concepts of the Galois field and the AES algorithm along with the quantified cryptographic strength of the transformations and the encoding Tamil texts. It also uses a Python programming environment with libraries that include the Galois (which allows us to perform Galois field mathematical operations), the itertools module and its groupby method, and the collections module and its counter tool.

**Figure 8:**
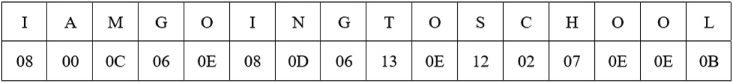
An encoded English sentence (in hex forms)

In addition, the method uses the English plaintext “I am going to school” and its Tamil-translated versions to study the effects of the proposed HRT-Grid and GFT-Grid in the application of AES and show the cryptographic strength of the Tamil text. Five versions of the Tamil translation of the English plaintext “I am going to school” are included in the proposed analysis. Figures 3, 4, and 8 explain the assignment of weights to the letters of English alphabet, their representation in hexadecimal format, and the pre-round transformation of a block of texts that carry the message of “I am going to school,” respectively. The encoding of its Tamil counterpart is discussed in the subsections, Tamil alphabet to HRT-Grid, HRT-Grid to Galois field, and Computational framework.

### Tamil alphabet to HRT-Grid

The first task is to model the relationship between the Tamil language and the prime factors of the Hardy-Ramanujan number 1,729. The Tamil alphabet has 247 letters, as shown in Figure 7. It has 12 vowels (first row; columns 2–13), 18 consonants (first column; rows 2–19), and a special character. The remaining 216 characters are formed by combining the sounds (phonics) of the 12 vowels and 18 consonants. Hence, to mathematically model the Tamil language, the vowels set V and the consonants set C are, respectively, defined as follows:

$$V={\left\{\varphi ,அ,ஆ\;\ldots ,ஔ\right\}}_{13},and$$

$$C={\left\{\varphi ,\mathrm{க்},\mathrm{ங்},\;\ldots ,\mathrm{ன்}\right\}}_{19}\text{.}$$

Note that both the vowel and consonant sets include a null element φ. Figure 7 represents the product set V×C that has all the ordered pairs of the elements of the vowel set V and the consonants set C of the Tamil alphabet. Hence, it has 247 tuples <v,c>, where v∈V and c∈C. We can now reveal that the first pair <φ,φ> of the product set forms the special Tamil character ஃ (akh), which is called the Ayutha Ezhuthu (tool letter) in Tamil—it could have been used as a tool to mold other letters. Hence, a one-to-one sound function f:V×C→X can be defined for the Tamil letters as follows:

(Equation 3) f(v,c)=x,

where v∈V, c∈C, and x∈X. The set X represents the Tamil alphabet that consists of the 247 characters, as shown in Figure 7. The product set V×C has a dimension of 13×19 that is two of the three prime factors of the Hardy-Ramanujan number 1,729; hence, the domain V×C of the Tamil sound function f is called the HRT-Grid in this article. Let us now algebraically expand the HRT-Grid into two square grids as follows:

and (Equation 4) $$13\times 19=\left(16-3\right)\times \left(16+3\right)=16^{2}-3^{2}\text{,}$$

(Equation 5) $$13\times 19+3^{2}=16^{2}\text{.}$$

This describes that the number of letters in the Tamil alphabet (or the number of elements in the HRT-Grid) plus the number of digits in the set Y={1,2,…,9} is equal to the maximum number of 8-bit integers that we could have. The square grid of 9 digits and the square grid of 256 integers are, respectively, called the APT-Grid and the GFT-Grid. The modeling of the GFT-Grid and the revelation of a cryptographic connection between Tamil letters and the Galois field are presented below.

### HRT-grid to Galois field

Now, suppose we have a set of 4-bit integers that are represented by the hexadecimal numbers $H={\left\{0,1,\ldots ,9,A,B,C,D,E,F\right\}}_{16}$; we can then define a product set $Q=H\times H={\left\{<0,0>,<0,1>,\ldots ,<F,F>\right\}}_{256}$ that consists of all 256 ordered pairs of the 16 elements of the set H. It defines an 8-bit integer by pairing 4-bit integers. We can define a one-to-one mapping g:X∪Y→H×H,

(Equation 6) $$g\left(y\right)=h_{1}h_{2}\text{,}$$

where y∈X∪Y and $h_{1}$ and $h_{2}$ are hexadecimal (hex) numbers that form 8-bit integers $h_{1}h_{2}$. The set Y represents the set of 9 digits Y={1,2,…,9}. The one-to-one mapping between y and $h_{1}h_{2}$ may be defined in many ways depending on whether y∈X or y∈Y. One way to generate this mapping is presented in Figure 5, which uses the following one-to-one mapping for y∈Y:

$$\begin{array}{r} g\left(1\right)=00;\;g\left(2\right)=1F;\;g\left(3\right)=3E;\;g\left(4\right)=5D;\;g\left(5\right)=7C;g\left(6\right)=9B;\;g\left(7\right)=BA\text{;}\\ g\left(8\right)=D9;\;g\left(9\right)=F8\text{.} \end{array}$$

In mathematics, a field is an infinite domain of elements (or observations) on which two pairs of operations—addition/subtraction and multiplication/division—are performed, with the exception of not allowing division by zero. In practical applications, the data domains are generally finite; hence, a finite field must be defined. It is well known that for a field to be finite, the number of elements in the field must satisfy $a^{p}$, where the base a and the exponent p are a prime number and a positive integer, respectively. In this case, the finite field is called the Galois field and denoted by GF $\left(a^{p}\right)$. In cryptography, the AES technique uses the finite field GF $\left(2^{8}\right)$ since 2 is a prime number and the elements are considered 8-bit numbers; hence, the number of elements in the Galois field GF $\left(2^{8}\right)$ is 256 (or $16^{2}$). In the proposed model, the one-to-one function g maps every element y of the HRT-Grid and the APT-Grid to an 8-bit integer $h_{1}h_{2}$ that lies in the GFT-Grid. Hence, the GFT-Grid has 256 characters (or hex numbers); thus, it forms a Galois field of GF $\left(2^{8}\right)$ with a multiplication operator ⊗ and an addition/subtraction operator ⊕ over the irreducible polynomial $x^{8}+x^{4}+x^{3}+x+1$. The multiplication and addition tables of this Galois field have a 256×256 dimension. For example, a Tamil letter in the multiplication table is a multiplication of another two Tamil letters in the table over the irreducible polynomial $x^{8}+x^{4}+x^{3}+x+1$:

நா⊗ம்=6E⊗87=0E=க்

நா⊕ம்=6E⊕87=E9=றீ

Hence, we can generate a substitution box (called S-Box as in AES algorithm) for Tamil language encryption as an encoder, to be integrated in the AES’s encryption flow. The GFT-Grid in Figure 5 is the S-Box that is used in this article; however, as stated earlier, there are many possible assignments that will lead to a more secure framework if the adapted assignment is not disclosed. These assignments may also be used for authentication and integrity of the messages. As per the concept of the Galois field, the multiplication of the two Tamil letters நா and ம் is mapped back to the Tamil letter க் in the Galois field over the irreducible polynomial $x^{8}+x^{4}+x^{3}+x+1$. Similarly, the addition of them also mapped back to the letter றீ in the Galois field. There are 256 pairs of Tamil letters, along with nine representable digits, mapped to the same letter; hence, given a letter in the ciphertext, it is difficult to perform a brute force attack to recover its corresponding pair in the plaintext.

In the AES encryption, these Galois field operations are performed multiple times at multiple steps and multiple rounds in the encryption flow (forming a nested operation); therefore, given a letter in a ciphertext, it is very difficult to traverse back in the encryption flow and recover the plaintext of the ciphertext. This can be easily shown by the following finite field operations:

(ந⊗ன்)⊗றி(6D⊗F2)⊗E8=A3⊗E8=44=ட்,and

(ந⊕ன்)⊕றி(6D⊗F2)⊕E8=9F⊕E8=BA=7(Saturn).

As per the concept (associative law) of the Galois field, the multiplication of the three Tamil letters ந, ன், and றி is mapped back to the Tamil letter ட் in the Galois field over the irreducible polynomial $x^{8}+x^{4}+x^{3}+x+1$. Similarly, the addition of them also mapped back to the digit 7 (that represents the astrological planet Saturn) in the Galois field. Due to this iterative application of the Galois field operators, combined with many-to-one mapping, it is more difficult for adversaries to perform a brute force attack on the Tamil ciphers than the English ciphers to recover their plaintexts. Hence, the addition of modules to generate confusion and diffusion properties with the propagation effect makes it more difficult for adversaries to attack.

### Computational framework

**Figure 9:**
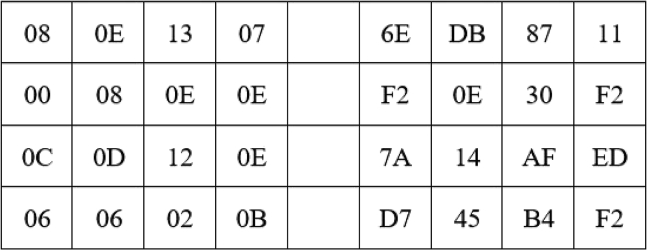
The transformation of the ciphertexts to states (in hex)

The computational framework of the proposed method, presented in Figure 1, provides a simulation architecture that helps systematically compile the presented mathematical approaches to model and simulate the cryptographic modules for encrypting English and Tamil texts using the AES algorithm. This computational framework shows two separate streams of simulation modules, the English-based AES simulation (the top stream) and the Tamil-based AES simulation (the bottom stream) to develop encryption flows. It also shows a common module, “Randomness measure,” that helps calculate the runs test scores and compare the ciphers of English and Tamil texts. The English-based AES simulation takes an English text and passes it through an encoder module and the AES module. The first module provides a simple encoder that allows the English text to be transformed to a hex state. It can be treated as the pre-round transformation of the AES algorithm. As stated earlier, the example of English text that is considered in this article is “I am going to school,” and it is presented in Figure 2. This figure also presents five versions of Tamil language texts that deliver the same message. The state output of the first module is delivered to the AES module, which then applies Sub-Bytes, ShiftRows, and MixColumns transformations. The ciphertext outputs of these transformations are then given to the “Randomness measure” module to calculate their runs test scores. In contrast, the Tamil-based AES simulation takes a Tamil text and passes it through five modules: Translate, HRT-Grid, GFT-Grid, and AES. The Translate module takes an English text and translates it to a Tamil text that delivers the same message as the English text. Alternatively, a Tamil text can be directly fed into the next module (i.e., the HRT-Grid module) without using the Translate module. Figure 6 shows some examples of the Tamil texts that provide the same meaning as “I am going to school.” The Tamil text is then delivered to the HRT-Grid module, which allows a one-to-one mapping to an index of the HRT-Grid. The output of this module is then delivered to the GFT-Grid module that provides the GFT-Grid and an S-Box to encode the Tamil text by utilizing the Tamil alphabet and the APT-Grid. This encoded text is then delivered to the AES module, as performed earlier, which then applies Sub-Bytes, ShiftRows, and MixColumns transformations. The intermediate ciphertext outputs are then given to the “Randomness measure” module to calculate their runs test scores. These two simulations allow us to compare the cryptographic strengths of various English and Tamil texts at every step of the encryption flow of the AES. The state representations of the English and Tamil encoders are presented in Figure 9. We can see a significant redundancy (non-randomness property) in the encoded ciphertext of English texts compared with the Tamil texts.

### Conclusions

This article showed a mathematical relationship between the Tamil language and the Hardy-Ramanujan number. It also showed that Tamil letters, combined with the digits 1 to 9, are members of a Galois field of GF $\left(2^{8}\right)$ with an irreducible polynomial of degree 8. This article also presented a TL encoder (a pre-round encryption module) that transforms the Tamil texts into hex states. This encoder can replace the pre-round transformation of the AES when the goal is the encryption of Tamil texts. The experimental analysis showed that the TL encoder can induce increased randomness in the intermediate ciphers that are the outputs of the Sub-Bytes, ShiftRows, and MixColumns transformations of the AES encryption flow. Therefore, based on this empirical study, the Tamil language could offer cryptographic strengths to AES-based confidentiality protection and enhance cybersecurity requirements. It can also support message authentication and integrity requirements. Also, note that the goal was to reveal the hidden HRT-Grid and GFT-Grid properties of the Tamil language and show its cryptographic strengths; hence, Unicode values are not used. However, one could linearly shift the values of the grids relatively and establish a significant correspondence. Therefore, our future research on this topic will focus on the modification of the model to meet the Unicode requirements. The future research will also focus on the adaptive placement of the elements of the APT-Grid on the GFT-Grid and its contributions to the confidentiality protection.

## Data Availability

The data (i.e., the Tamil texts) used in this study are already presented in the paper. The Python modules that are used to produce the results are included in the supplemental information.

## Supplemental information

Document S1. Figures S1 and S2

Document S2. Article plus supplemental information
