# Supplementary material for: Modeling and simulation of a Tamil language encoder for advanced encryption technologies
Source: Patterns (N Y). 2023 May 3;4(7):100740. doi: 10.1016/j.patter.2023.100740 (PMC10382943; doi:10.1016/j.patter.2023.100740)
Supplement: Document S1. Figures S1 and S2 [file mmc1.pdf]

Patterns, Volume *4*

Supplemental information

Modeling and simulation of a Tamil language encoder for advanced encryption technologies

Shan Suthaharan

```
import math
from itertools import groupby
from collections import Counter

stateE = ["08", "00", "0C", "06", "0E", "08", "0D", "06", "13", "0E", "12", "02", "07", "0E", "0E", "0B"]

binStringE = bin(int(stateE[0], 16))[2:].zfill(8)
for ii in range(1, 16):
    tmpE = bin(int(stateE[ii], 16))[2:].zfill(8)
    binStringE = binStringE+tmpE

print(binStringE)
m1 = binStringE.count('0')
m2 = binStringE.count('1')
print(m1,m2)

ER2 = ((2*m1*m2)/(m1+m2))+1
SD2 = math.sqrt((2*m1*m2*(2*m1*m2-m1-m2))/(((m1+m2)**2)*(m1+m2-1)))

cE = Counter(k for k, g in groupby(binStringE))
runsE = cE['0']+cE['1']
Z2 = abs(runsE-ER2)/SD2
print(Z2)

%Here the null hypothesis H0: The sequence is random
%The output of Z2 should be 3.1016 which is greater than 1.96, hence, we can reject H0 with 95% confidence.
%Therefore, "The␣English␣text␣bit␣sequence␣is␣NOT␣random")

%The bit sequence is:
%000010000000000000001100000001100000111000001000000011
%010000011000010011000011100001001000000010000001110000
%11100000111000001011
```

Figure S1: Runs test that validates the randomness of English

```
import math
from itertools import groupby
from collections import Counter

stateT = ["6E", "F2", "7A", "D7", "DB", "0E", "14", "45", "87", "30", "AF", "B4", "11", "F2", "ED", "F2"]

binStringT = bin(int(stateT[0], 16))[2:].zfill(8)
for ii in range(1, 16):
    tmpT = bin(int(stateT[ii], 16))[2:].zfill(8)
    binStringT = binStringT+tmpT

print(binStringT)
n1 = binStringT.count('0')
n2 = binStringT.count('1')
print(n1,n2)

ER1 = ((2*n1*n2)/(n1+n2))+1
SD1 = math.sqrt((2*n1*n2*(2*n1*n2-n1-n2))/(((n1+n2)**2)*(n1+n2-1)))

cT = Counter(k for k, g in groupby(binStringT))
runsT = cT['0']+cT['1']
Z1 = abs(runsT-ER1)/SD1
print(Z1)

%Here the null hypothesis H0: The sequence is random
%The output of Z1 should be 0.287 which is less than 1.96, hence, we can accept H0 with 95% confidence.
%Therefore, "The␣Tamil␣text␣bit-sequence␣is␣random")

%The bit sequence is:
%011011101111001001111010110101111101101100001110000101
%000100010110000111001100001010111110110100000100011111
%00101110110111110010
```

Figure S2: Runs test that validates the randomness of Tamil
